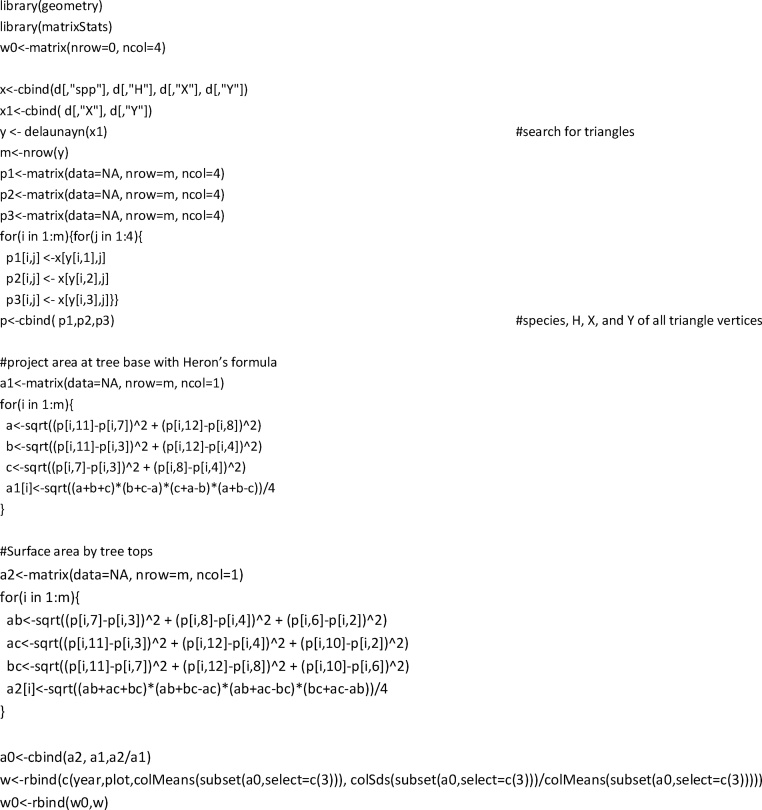# Assessing stand species and structural diversity at neighbourhood scale

**DOI:** 10.1016/j.mex.2018.02.002

**Published:** 2018-02-21

**Authors:** Hua Yang, Rongzhou Man

**Affiliations:** a State Forestry Administration Key Laboratory of Forest Resources & Environmental Management, Beijing Forestry University, China; b Ontario Forest Research Institute, Ontario Ministry of Natural Resources and Forestry, Canada

**Keywords:** Neighbourhood diversity indices, Tree neighbours, Spatial complexity/heterogeneity, Spatial scale, Stand structure

## Abstract

Forest diversity assessments are typically conducted at stand scale. This traditional diversity assessment may provide substantial insight into overall stand structure but is limited with respect to describing within-stand variation, an important aspect of stand diversity. This article describes a method for assessing species and structural diversity at within-stand, neighbourhood scale.

• Nearest neighbours are determined from mapped tree locations in field survey plots.
• R codes (provided in appendices) are used to assist with determining species and structural diversity indices at a neighbourhood of 4 trees (a subject tree and the 3 nearest neighbours).
• Neighbourhood structural diversity indices are compared against structural complexity index (SCI) in capturing within-stand variation.
• Neighbourhood diversity indices, especially in managed stands, are useful for capturing spatial variation in species and structural diversity.

## Graphical abstract

Neighbourhood diversity values and spatial variations are derived from mapped tree locations in field survey plots.

**Figure:**
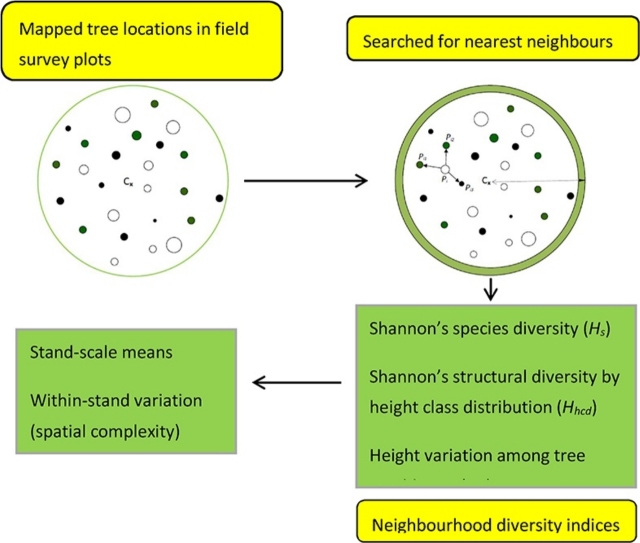

## Method details

The Shannon’s diversity index (*H*′) is commonly used to assess stand scale diversity [1]:

(1) $$H^{\prime }=-\overset{r}{\underset{i=1}{\sum }}p_{i}\text{ln}p_{i}$$

Where *p_i_* is the proportion of individuals (or their basal area, crown cover, foliar cover, or biomass) in the *i*th species (or the diameter and height class) [2]. Man and Yang [[3], [4]] expand this stand scale assessment to evaluate species and structural diversity at neighbourhood scale using stem mapping data.

(2) $$H_{s}=-\frac{1}{n}\overset{n}{\underset{i=1}{\sum }}\overset{r}{\underset{j=1}{\sum }}p_{ij}\text{ln}p_{ij}$$

**Fig. 1:**
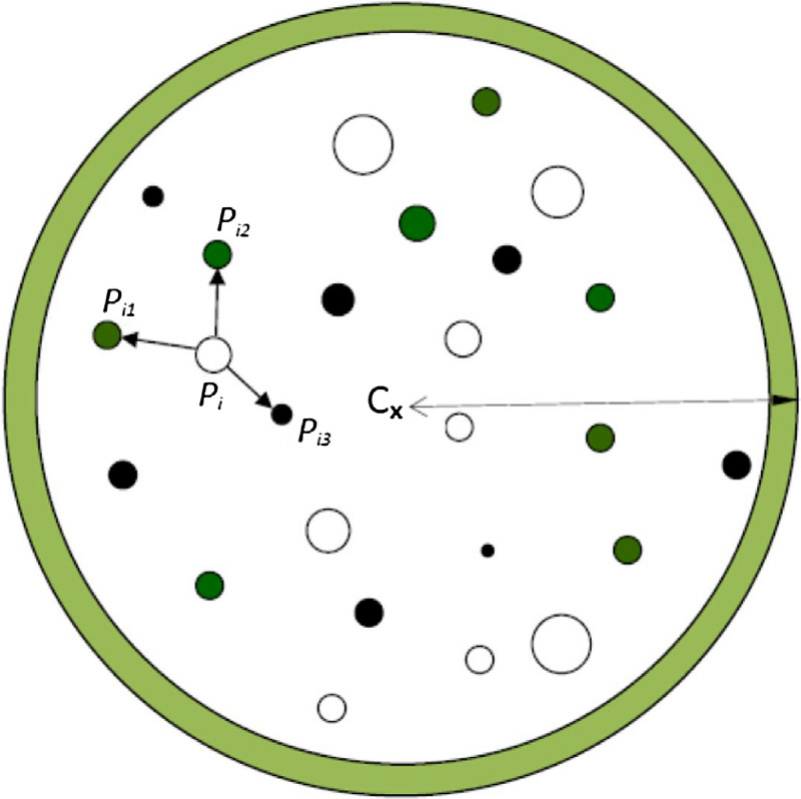
Example of a stem-mapped plot (centred at C) showing the locations of subject tree *P_i_*. (species A) and its 3 nearest neighbours, *P_i1_*, *P_i2_*, and *P_i3_* (2 species B and 1 species C). Size of circles is relative to size of tree; colours indicate species. Trees in the shaded area surrounding the plot edge are not included in estimates of stand-scale means (Adapted from Fig. 1 in [3]).

Therefore, by measuring *H*′ for all subject trees (n) in a stand, the stand mean neighbourhood species diversity (*H_s_*) can be found. A neighbourhood consists of *i*th subject tree and its *k* nearest neighbours (Fig. 1). Where *r* is the number of tree species (*r *≤ *k* + *1*) and *p_ij_* is the proportion of trees in *j*th species within a neighbourhood of *k* + 1 trees. The neighbourhood structural diversity indices by height class distribution (*H_hcd_*) or height variation (*S_hv_*) can be derived similarly. The within-stand spatial variation of neighbourhood diversity depends on assessment scale and therefore neighbourhood size and can be evaluated with the coefficients of variation (CV) among tree neighbours.

The determination of neighbourhood diversity requires identifying tee neighbours of desired size for all subject trees in the stand. The algorithm nn2 within the R package RANN version 2.4.1 [5] is used to search for the nearest neighbours based on x, y coordinates of trees (Appendix A). Before estimating stand means, two adjustments are required: 1) to eliminate the possible influence of false neighbours at plot edges (Fig. 1), all subject trees and associated tree neighbours in boundary zone are removed; 2) to take a random sub-sample of the tree neighbours to reduce possible overlap among them (the process repeats 10 times to increase precision of estimated stand means) (Appendix A).

Large tree neighbours provide better approximation of stand scale assessments, but a neighbourhood of 4 trees (a subject tree and the 3 nearest neighbours) captures considerable stand scale information, and assists comparisons with other small scale diversity indices (such as SCI) and stand structure reconstruction research, composition interpretations of mixed tree plantations, and field data collection when stem mapping data are not available [3].

## Comparison with structural complexity index

**Fig. 2:**
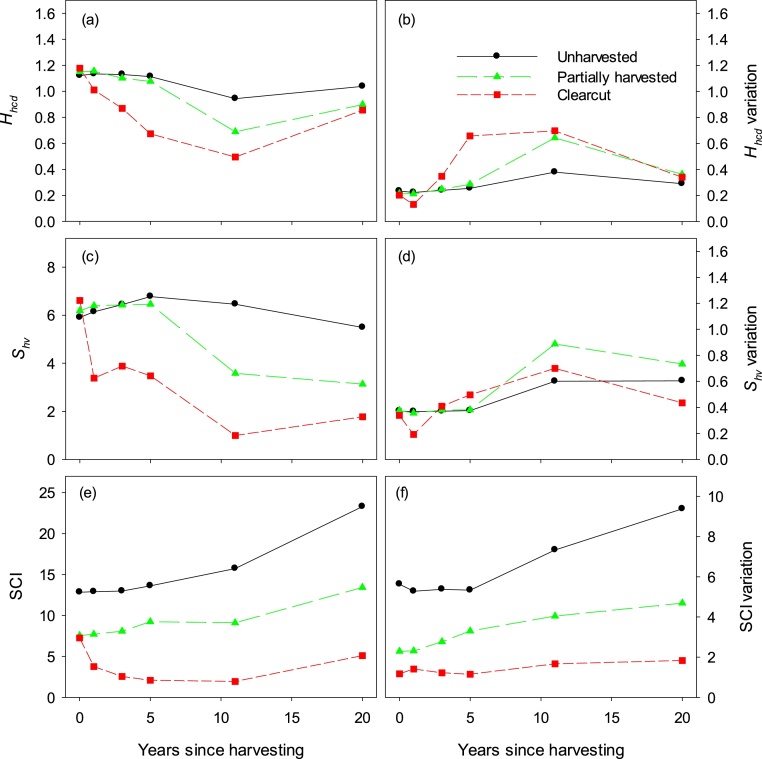
Neighbourhood structural diversity values and within-stand spatial variations (coefficients of variation) of overstory trees by harvesting treatment and time since harvest: (a, b) Shannon structural *H′* by 2-m height class, (c, d) neighbourhood height variation, and (e, f) structural complexity index (SCI) (Adapted from Fig. 2 in [4]).

Structural complexity index (SCI) [6], a stand structural diversity assessed at neighbourhood scale, is calculated using Delaunayn triangulation function in the R package geometry version 0.3–6 [7] (Appendix B). All three small-scale structural diversity indices (*H_hcd_*, *S_hv_*, SCI) are similar in post-harvesting among-treatment differences (Fig. 2). The unharvested treatment had the highest structural diversity values, whereas clearcut had the lowest. Within-stand variations (spatial complexity), however, are higher in harvested (partially harvested and clearcut) than in unharvested treatments. Comparatively, SCI is less useful for capturing within-stand variation among harvested and leave strips at reduced stand densities by harvest.

## Figures and Tables

**Figure:**
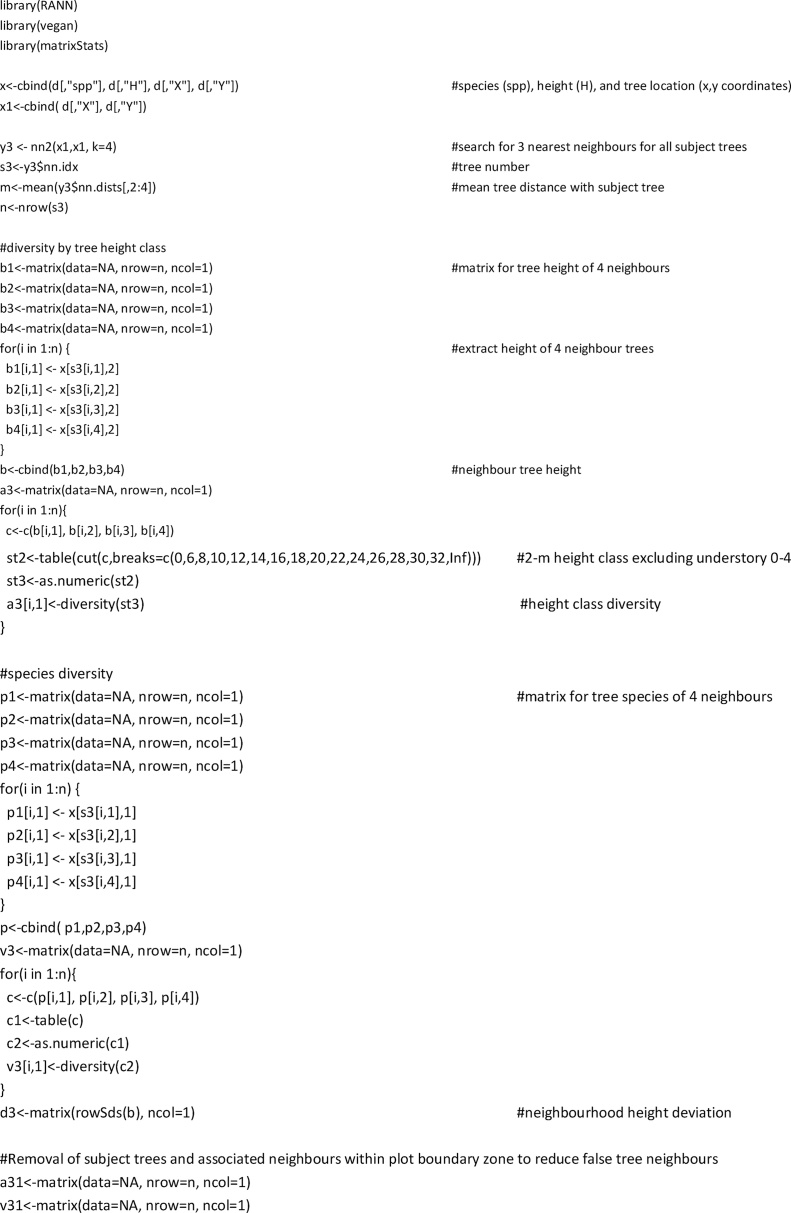


**Figure:**
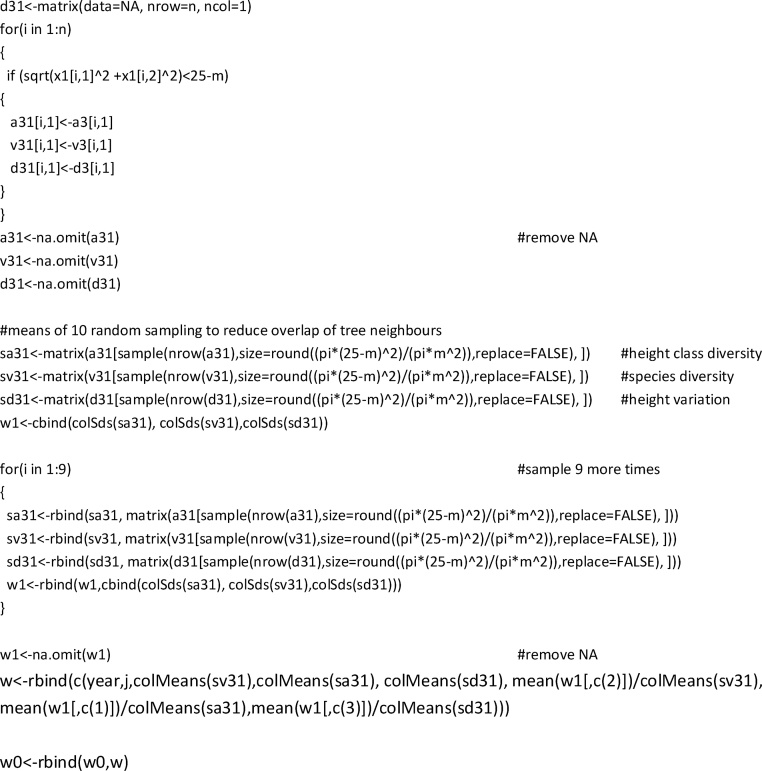


**Figure:**